# Inducible targeting of CNS astrocytes in Aldh1l1-CreERT2 BAC transgenic mice

**DOI:** 10.12688/f1000research.10509.1

**Published:** 2016-12-30

**Authors:** Jan Winchenbach, Tim Düking, Stefan A. Berghoff, Sina K. Stumpf, Swen Hülsmann, Klaus-Armin Nave, Gesine Saher

**Affiliations:** 1 Department of Neurogenetics, Max Planck Institute of Experimental Medicine, Göttingen, Germany; 2 Clinic for Anesthesiology, Research Group Central Respiratory Control, University Medical Center Göttingen, Experimental Neuroanesthesiology, Göttingen, Germany; 3 Center Nanoscale Microscopy and Molecular Physiology of the Brain (CNMPB), Göttingen, Germany

**Keywords:** Astrocyte, Bergman glia, inducible Cre recombinase, tamoxifen, neuroscience

## Abstract

*Background: *Studying astrocytes in higher brain functions has been hampered by the lack of genetic tools for the efficient expression of inducible Cre recombinase throughout the CNS, including the neocortex. *Methods: *Therefore, we generated BAC transgenic mice, in which CreERT2 is expressed under control of the *Aldh1l1* regulatory region. *Results: *When crossbred to Cre reporter mice, adult Aldh1l1-CreERT2 mice show efficient gene targeting in astrocytes. No such Cre-mediated recombination was detectable in CNS neurons, oligodendrocytes, and microglia. As expected, Aldh1l1-CreERT2 expression was evident in several peripheral organs, including liver and kidney. *Conclusions: *Taken together, Aldh1l1-CreERT2 mice are a useful tool for studying astrocytes in neurovascular coupling, brain metabolism, synaptic plasticity and other aspects of neuron-glia interactions.

## Introduction

Cre-mediated recombination of target genes in adult astrocytes requires the use of an inducible expression system, because many promoters of the astrocyte lineage are also active in multipotential neural stem cells in the subventricular and subgranular zones ( Christie *et al.*, 2013). Thus, transgenic mouse lines have been generated for tamoxifen-inducible Cre recombination of target genes in mature astrocytes ( Chow *et al.*, 2008; Ganat *et al.*, 2006; Hirrlinger *et al.*, 2006; Mori *et al.*, 2006; Slezak *et al.*, 2007). However, none of them achieves sufficient recombination to study the function of genes in the majority of cortical and spinal cord astrocytes.

The aldehyde dehydrogenase 1 family member L1 (Aldh1l1), also known as 10-formyltetrahydrofolate dehydrogenase (EC 1.5.1.6), converts 10-formyltetrahydrofolate to tetrahydrofolate and CO _2_ together with the reduction of NADP ^+^ ( Kutzbach & Stokstad, 1971). The *Aldh1l1* gene is expressed in a subset of radial glia in the midline of the embryonic CNS ( Anthony & Heintz, 2007) and neuronal precursors ( Foo & Dougherty, 2013). By transcriptional profiling in postnatal brain, *Aldh1l1* was identified to be specifically expressed in astrocytes ( Cahoy *et al.*, 2008), which increase *Aldh1l1* expression about tenfold with maturation ( Zhang *et al.*, 2014). To date, Aldh1l1 is regarded a pan-astrocyte marker, as determined in BAC transgenic mice with a fluorescent reporter protein or constitutive Cre expression under control of the *Aldh1l1* promoter ( Heintz, 2004; Yang *et al.*, 2011). Therefore, we selected the *Aldh1l1* regulatory region and a similar BAC transgenic strategy to target transgenic expression of CreERT2 to mature astrocytes.

## Results and discussion

### Efficiency of recombination in astrocytes of Aldh1l1-CreERT2 transgenic mice

**Figure 1.**
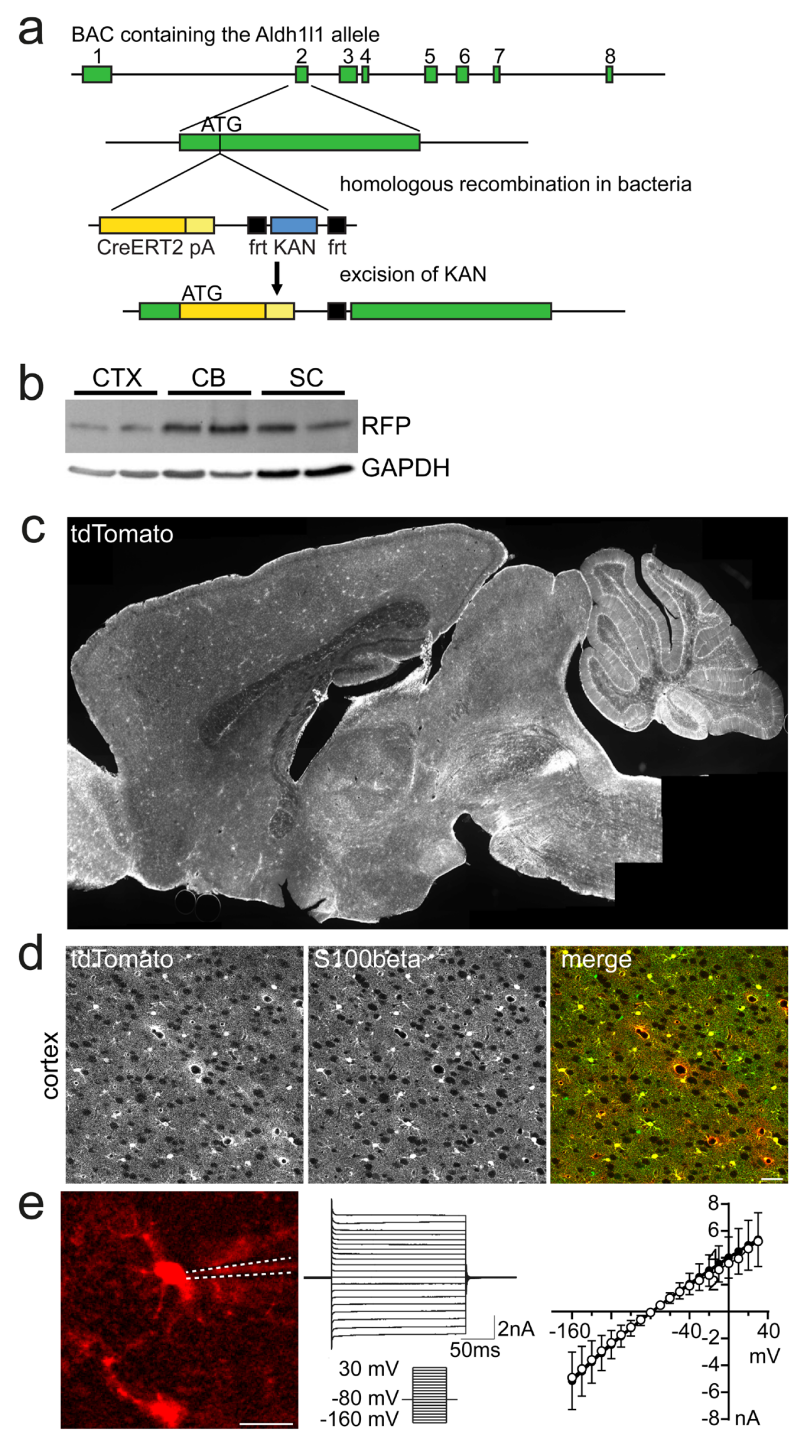
The Aldh1l1 BAC transgene efficiently targets CNS astrocytes. **a**) Scheme of the cloning strategy of Aldh1l1-CreERT2 BAC transgene. **b**) Immunoblot detecting RFP (tdTomato) in cortex (CTX), cerebellum (CB) and spinal cord (SC) lysates of two animals each, as indicated. GAPDH shows comparable loading of protein. **c**) Direct fluorescence of the Cre-reporter tdTomato in sagittal sections of Aldh1l1-CreERT2*ROSA26-Tdto mice. **d**) Immunolabeling of the astrocyte marker S100beta in the cortex reveals almost complete overlap with the tdTomato Cre reporter in astrocytes. Scale, 20 µm. **e**) CCD camera image of a tdTomato positive astrocyte with the position of the patch pipette outlined as dashed lines (scale, 20 µm, left) that showed a typical passive response to the voltage step protocol (middle). The IV-curve of this cell is shown (right panel, open circles) together with the averaged IV curve of all 18 analyzed cells (mean ± sd).

We generated Aldh1l1-CreERT2 transgenic mice by inserting a CreERT2 cassette ( Sauer, 1994) under control of the *Aldh1l1* promoter in a murine BAC (BAC RP23-7M9). Targeting the first coding exon of Aldh1l1 by homologous recombination, we substituted the open reading frame of exon 2 with the CreERT2 cDNA ( Figure 1a). Three lines of BAC transgenic mice were obtained by pronuclear injection, and crossbred with the Cre reporter mice ROSA26-Tdto or ROSA26-Eyfp ( Madisen *et al.*, 2010; Srinivas *et al.*, 2001). Based on the degree of expression, one of the three lines of Aldh1l1-CreERT2 mice was selected for detailed characterization of double-transgenic offspring.

First, we determined the leakiness of reporter expression in adult Aldh1l1-CreERT2 mice. After corn oil injections in Aldh1l1-CreERT2*ROSA26-Tdto mice, we found very few labeled cells (less than 5 per section), demonstrating that the inducible Cre system operates tightly. In parallel experiments, adult Aldh1l1-CreERT2 mice were analyzed 7 days after tamoxifen induction. Sagittal brain sections revealed numerous tdTomato Cre reporter expressing cells, which in the forebrain exhibited the typical morphology of protoplasmic astrocytes ( Figure 1). Co-labeling revealed that almost all S100beta (S100 calcium-binding protein B) positive cells in hippocampus and cerebral cortex expressed tdTomato ( Figure 1, Table 1).

**Table 1.** Efficiency and specificity of Aldh1l1-CreERT2 mediated recombination in brain. Efficiency and specificity of inducible Cre mediated recombination in adult Aldh1l1-CreERT2 mice crossbred with Cre reporter ROSA26-Tdto or ROSA26-Eyfp. For each value shown (average percentage), cells were counted on eight confocal images and two sections for each of n=4 animals. Efficiency is expressed as percent Cre reporter positive cells of all S100beta labeled cells. Specificity is expressed as percentage of all Cre reporter positive cells that lack immuno-labeling for S100beta.

Region	Marker	Co-labelled cells (%)	Number of analyzed cells
*Efficiency*			
Cortex (astrocytes)	tdTomato/S100beta EYFP/S100beta	92 ± 2 62 ± 2	1868 2038
Cerebellum (Bergman glia) Corpus callosum Fimbria	EYFP/S100beta tdTomato/S100beta tdTomato/S100beta	89 ± 1 85 ± 1 94 ± 2	1460 713 145
*Specificity*			
Cortex (astrocytes)	S100beta neg./tdTomato S100beta neg./EYFP	12 ± 3 19 ± 3	1943 1553
Cerebellum (Bergman glia) Fimbria	S100beta neg./EYFP S100beta neg./tdTomato	6 ± 1 4 ± 1	1397 143

For comparison, when using a less sensitive EYFP Cre reporter line ( Srinivas *et al.*, 2001) in corresponding experiments, only two thirds of all S100beta positive cells in the cortex were also EYFP positive ( Table 1). Thus, although both Cre reporter lines were generated as a knock-in into the endogenous ROSA26 locus, the recombination efficacy achieved is clearly different, in agreement with previous reports ( Madisen *et al.*, 2010; Srinivas *et al.*, 2001). This finding illustrates the need to determine recombination efficiency individually for each combination of Cre allele and floxed target gene.

To characterize the identity of targeted cells functionally, we patched in total 18 tdTomato expressing cells in the cortex ( Figure 1e). As expected, all cells displayed the electrophysiological signature of mature astrocytes ( Grass *et al.*, 2004; Schipke *et al.*, 2001), with low input resistance (20.79 ± 9.26 mean MΩ ± sd; n=18) and negative resting membrane potential (-78.71 ± 3.22 mV).

**Figure 2.**
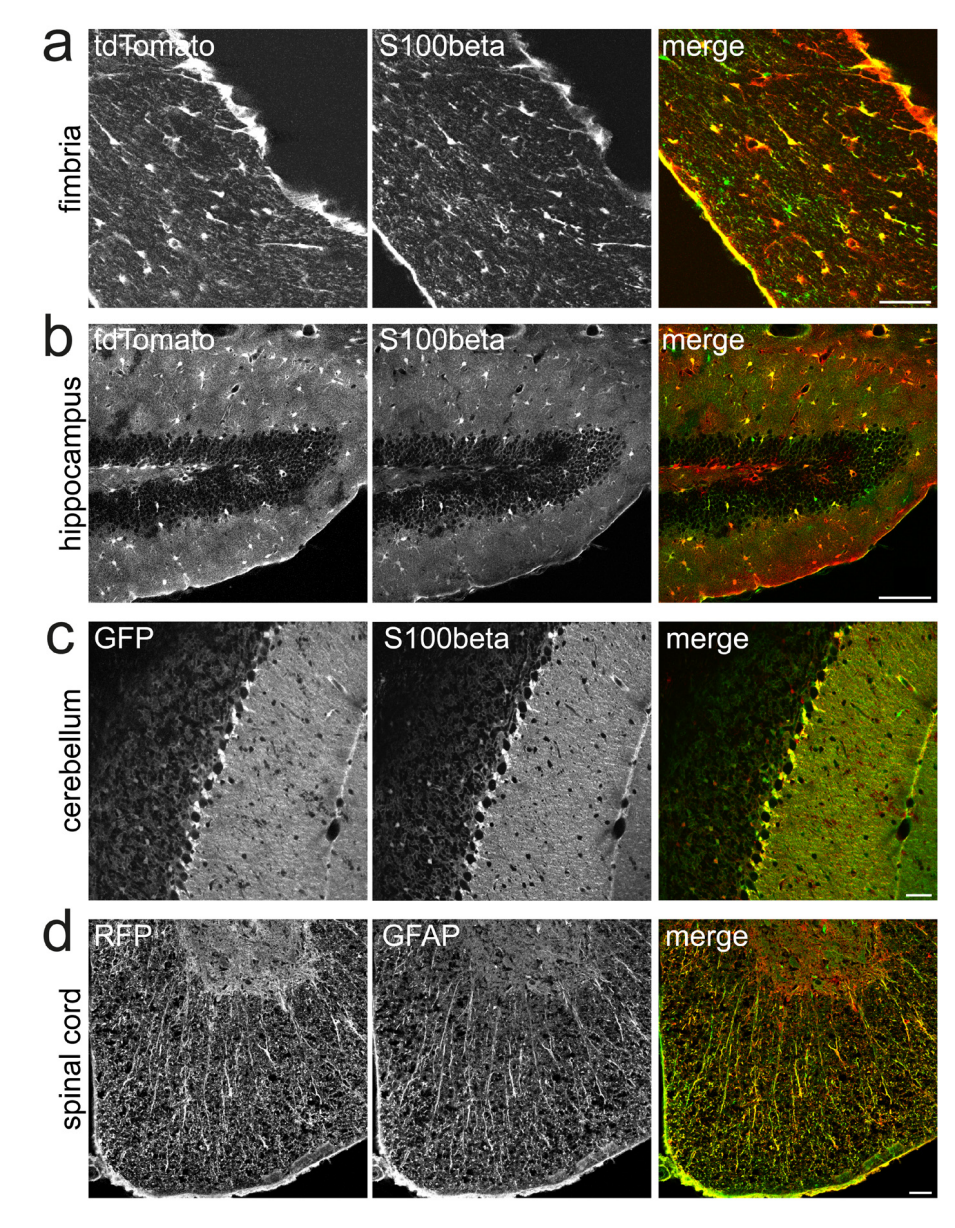
Inducible targeting of Bergman glia and white matter astrocytes. Co-immunolabeling of the astrocyte marker S100beta or GFAP with Cre reporter (direct tdTomato fluorescence, GFP anti EYFP or RFP anti tdTomato) in fimbria ( **a**), hippocampus ( **b**), cerebellum ( **c**) and spinal cord ( **d**) reveals almost complete overlap of the transgene with astrocytes. Scale, 50 µm.

The expression pattern of some astroglial marker proteins, such as GFAP (glial fibrillary acidic protein), differs between protoplasmic astrocytes in the cortex and fibrous astrocytes in white matter. We therefore assessed the efficacy of Cre recombination separately for the corpus callosum, fimbria, hippocampus and spinal cord. Again, in all these regions a large majority of astrocytes, as defined by S100beta or GFAP, expressed Cre reporter, e.g. 85±1% in the corpus callosum and 94±2% in the fimbria (n=3 animals) ( Figure 2, Table 1). Co-labeling with GFAP was not used for cell counts because of the protein’s low abundance in cell bodies which makes unequivocal quantification difficult.

In the cerebellum, a large fraction (89 ± 1%) of S100beta positive Bergman glia cells expressed the Cre reporter EYFP ( Figure 2c, Table 1). While 3.3 ± 0.3% of parvalbumin positive interneurons of the molecular layer expressed the tdTomato Cre reporter, none was double positive in corresponding experiments using the EYFP Cre reporter, confirming the sensitivity of the tdTomato reporter with a tendency for off-target recombination. Cre reporter expression was also observed in some neurons in the dentate gyrus and olfactory bulb, likely reflecting some recombination in adult neural stem cells in the subgranular and subventricular zone, followed by the migration of labeled progeny through the rostral migratory stream ( Figure 1c).

**Figure 3.**
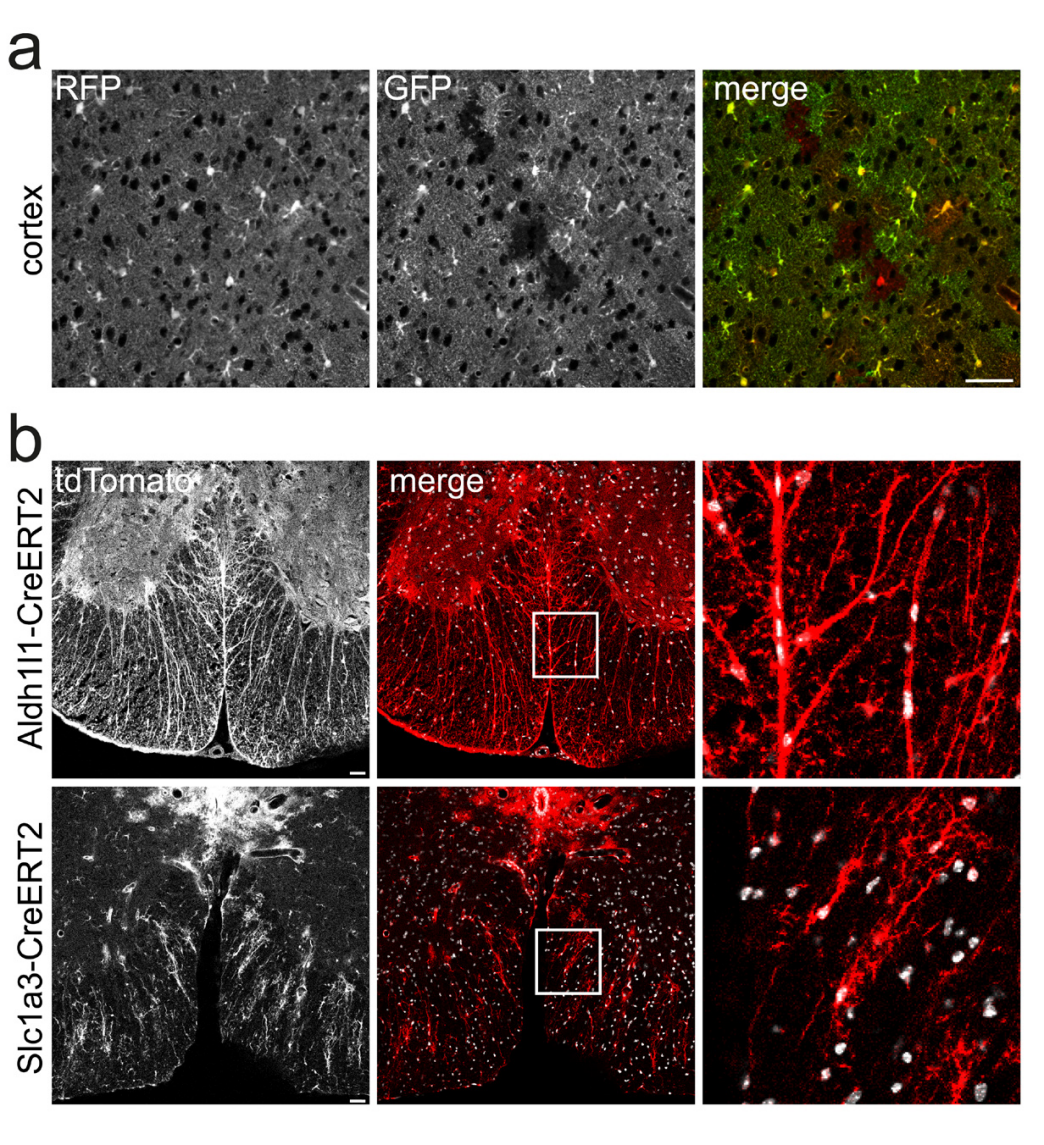
Comparison with other transgenes targeting astrocytes. **a**) Co-immunolabeling of the Cre reporter tdTomato (anti RFP) and EGFP (anti GFP) in triple transgenic mice (Aldh1l1-CreERT2*ROSA26-Tdto*Aldh1l1-Egfp) in cortical sections. Scale 50 µm. **b**) Direct fluorescence of the Cre-reporter tdTomato in spinal sections of Aldh1l1-CreERT2*ROSA26-Tdto and Slc1a3-CreERT2*ROSA26-Tdto transgenes. Scale 50 µm.

Next, we compared Aldh1l1-CreERT2 mediated recombination with the expression pattern of EGFP in Aldh1l1-Egfp transgenic mice, generated with a similar BAC based strategy ( Heintz, 2004). As expected, reporter and EGFP expression was nearly identical in the cortex, confirming the high efficiency of CreERT2 mediated induction of the tdTomato reporter ( Figure 3a).

Finally, in comparison with Slc1a3 (Glast)-CreERT2 ( Mori *et al.*, 2006), Aldh1l1-CreERT2 mediated recombination of the tdTomato reporter revealed nearly complete recombination of astrocytes in spinal cord white matter, whereas Slc1a3-CreERT2 mediated fluorescence appeared patchy ( Figure 3b).

### Cellular specificity of Cre expression

**Figure 4.**
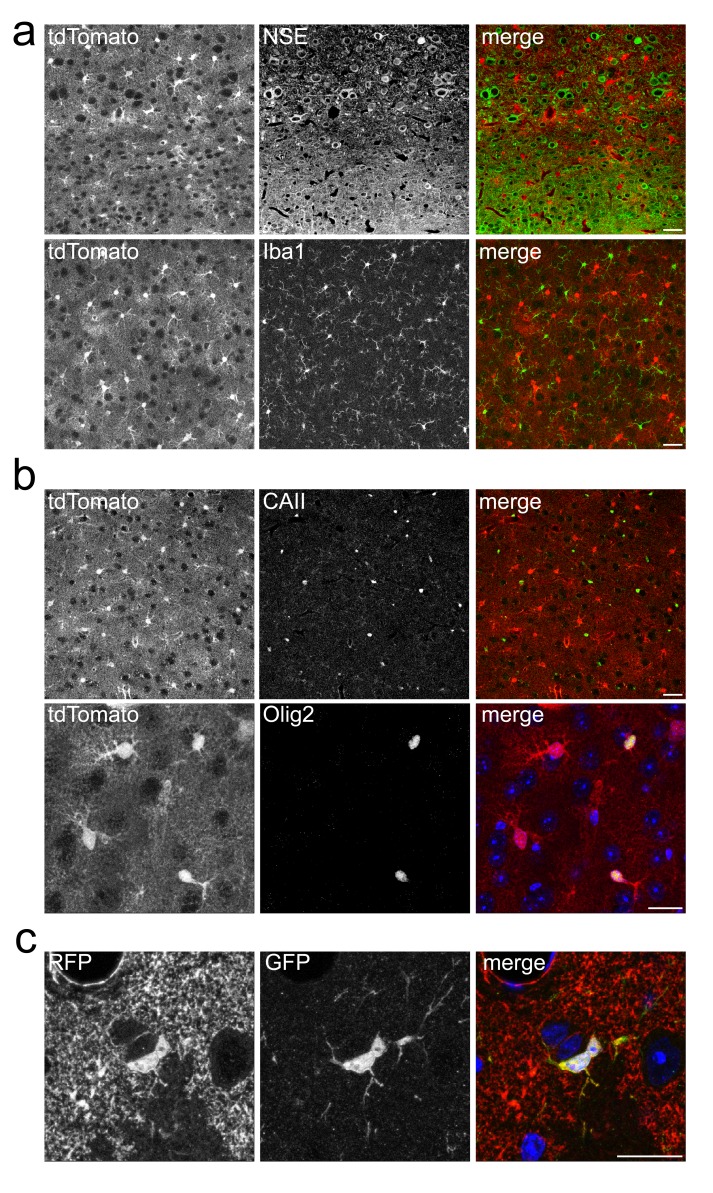
Specificity of Aldh1l1-CreERT2 mediated recombination. **a**) Direct fluorescence of the Cre-reporter tdTomato and immunolabeling of neurons (NSE) and microglia (Iba1) on cortical sections. Scale, 50 µm. **b**) Direct fluorescence of the Cre-reporter tdTomato and immunolabeling of mature oligodendrocytes (CAII, scale, 50 µm) and oligodendroglia (Olig2, scale, 20 µm). **c**) Co-immunolabeling of the Cre reporter tdTomato (anti RFP) and EYFP (anti GFP) in triple transgenic mice (Aldh1l1-CreERT2*ROSA26-Tdto*NG2-Eyfp) revealing co-labeling in a small fraction of cells. Scale, 20 µm.

Next, we tested the cell-type specificity of the Aldh1l1-CreERT2 transgene. Co-localization of tdTomato with markers for neurons (NSE, neuron specific enolase) or microglia (Iba1, ionized calcium binding adaptor molecule 1) was virtually absent ( Figure 4, Table 2). However, we observed a small fraction of Cre reporter positive cells co-localizing with Olig2 (oligodendrocyte lineage transcription factor 2), a transcription factor found in all oligodendrocyte lineage cells, including oligodendrocyte precursor cells ( Figure 4b). Similarly, in triple transgenic mice that additionally express EYFP under control of the endogenous NG2 (neural/glial antigen 2) promoter ( Karram *et al.*, 2008), we identified 3.4% of double labeled cells, presumably oligodendrocyte precursor cells based on their localization and morphology. However, co-localization with a marker of mature oligodendrocytes (CAII, carbonic anhydrase 2) was negligible 12d after tamoxifen injections, and did not increase in mice that were analyzed 27 weeks after recombination (tamoxifen induction at 16 weeks of age). This suggests that the small percentage of Aldh1l1-CreERT2 expressing NG2 glia does not give rise to oligodendrocytes. An independently generated line of Aldh1l1-CreERT2 mice ( Srinivasan *et al.*, 2016) shows some Olig1, Olig2, CNP and CAII but no NG2 expression, as determined by ribotag-dependent transcriptome profiling ( Sanz *et al.*, 2009). Whether this dissimilarity is caused by the different detection methods employed remains to be determined.

**Table 2.** Specificity of Aldh1l1-CreERT2 mediated recombination in brain. Specificity of inducible Cre mediated recombination in adult Aldh1l1-CreERT2*ROSA26-Tdto mice. For each value (average percentage), cells were counted on eight confocal pictures and two sections for each of n=4 animals. Specificity is expressed as percentage of cells that show Cre reporter expression of all cell type marker positive cells.

Region	Marker	Co-labelled cells (%)	Number of analyzed cells
Cortex	tdTomato/NSE tdTomato/Iba1 tdTomato/CAII tdTomato/EYFP (NG2)*	0 0.4 ± 0.3 1.8 ± 0.9 3.4 ± 0.8	1000 1157 443 1275
Cerebellum	tdTomato/Parvalbumin	3.3 ± 0.3	2504

*analyzed in triple transgenic mice (Aldh1l1-CreERT2*ROSA26-Tdto*NG2-Eyfp)

### Cre recombination in peripheral organs

**Figure 5.**
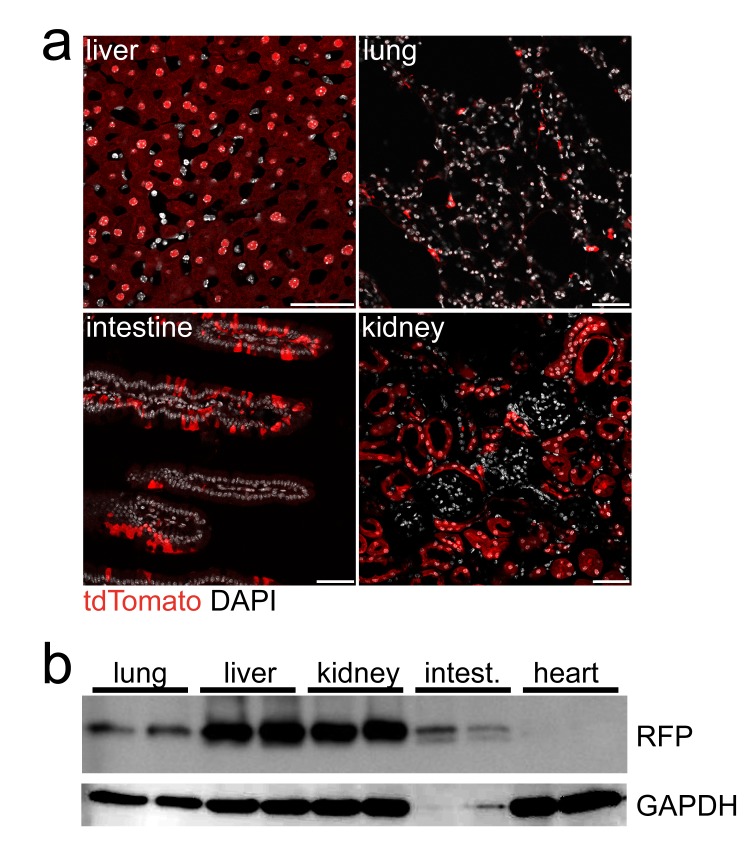
Recombination in peripheral organs. **a**) Direct fluorescence of the Cre-reporter in transgenic Aldh1l1-CreERT2*ROSA26-Tdto mice in liver, kidney, lung and intestine. Nuclei are shown in white (DAPI). Scale, 50 µm. **b**) Western blot detecting RFP (tdTomato) in lung, liver, kidney, small intestine, and heart, as indicated. GAPDH served as loading control.

Aldh1l1 is an enzyme of folate metabolism that is expressed in various peripheral organs ( Krupenko & Oleinik, 2002). In agreement, we detected Cre reporter expression in liver, kidney, lung, and small intestine by direct immunofluorescence and Western blotting ( Figure 5). Cre reporter was not detected in heart muscle.

Raw data generated or analyzed during the present study in a zipped file Click here for additional data file. Copyright: © 2016 Winchenbach J et al. 2016 Data associated with the article are available under the terms of the Creative Commons Zero "No rights reserved" data waiver (CC0 1.0 Public domain dedication).

## Conclusion

Aldh1l1 is a general marker for astrocytes within the CNS, and our new line of tamoxifen-inducible Aldh1l1-CreERT2 transgenic mice can be used to genetically target astrocytes in the mature CNS with high efficiency and specificity. When the corresponding genomic recombination in peripheral tissues is well tolerated, this line is suitable to study gene functions in astroglial cells of adult mice. Aldh1l1-CreERT2 mice will be made freely available upon request to the corresponding author.

## Methods

### Transgenic mice

All animal studies were performed at the Max Planck Institute of Experimental Medicine in compliance with the animal policies of the Max Planck Institute of Experimental Medicine and were approved by the German Federal State of Lower Saxony. All animals were housed in individually ventilated cages in groups of 3–5 mice per cage, kept in a room with controlled temperature (~23°C) under 12 h light/dark cycle and had access to food and water *ad libitum*. In addition to the newly generated inducible Aldh1l1-CreERT2 mouse line (see below), we used BAC transgenic Aldh1l1-Egfp mice ( Heintz, 2004), Slc1a3-CreERT2 mice (also called Glast-CreERT2; Mori *et al.*, 2006), and NG2-EYFP knock-in mice ( Karram *et al.*, 2008). As Cre reporter we used the ROSA26 flox-stop-flox-Tdtomato line (ROSA26-Tdto; Madisen *et al.*, 2010) and the ROSA26 flox-stop-flox-EYFP line (ROSA26-Yfp; Srinivas *et al.*, 2001). We used a total of 26 mice of both sexes at the age of 7–10 weeks unless otherwise stated (20 – 30 g body weight). All mice were analyzed as heterozygotes for the respective transgenic allele.

### Generation of Aldh1l1-CreERT2 mice

By PCR we introduced 50 bp of the *Aldh1l1* intron 1/ exon 2 sequence 5’ of the CreERT2 open reading frame. The bovine growth hormone poly A sequence (bGH pA), the frt (flippase recognition site) flanked kanamycin resistance cassette, and 50 bp of *Aldh1l1* genomic sequence was inserted into an Nhe1 site 3’ to the ERT2 sequence. The combined construct was introduced into exon 2 of the *Aldh1l1* gene on the BAC RP23-7M9 (BACPAC Resources of the Children's Hospital Oakland Research Institute in Oakland), in frame with the start ATG, by homologous recombination in bacteria (EL250) as described ( Lee *et al.*, 2001). Excision of the resistance cassette was done by arabinose induced flippase expression. The BAC insert was excised by Not I digestion and purified by size exclusion chromatography using a sepharose column. Pronucleus injection gave rise to 5 transgenic founder mice. Genotyping was done by PCR of purified tail genomic DNA under standard conditions with the primers (5’-3’, final concentration 0.25 µM) CAACTCAGTCACCCTGTGCTC and TTCTTGCGAACCTCATCACTCG amplifying the 3’ part of intron1 of the Ald1l1 gene to the 5’ part of the Cre open reading frame. Three out of five founder mice that were crossed with reporter mice showed expression in brain. Only one line (Aldh1l1-CreERT2 line 02) showed robust expression in forebrain astrocytic cells and minimal expression in other cell types of the brain. 

### Tamoxifen administration

Tamoxifen (Sigma, T5648) was dissolved in corn oil (Sigma, C8267) at a concentration of 7.5 mg/ml and injected intraperitoneally at 75 µg/g body weight on 5 consecutive days. We used a total of 26 mice of both sexes at the age of 7–10 weeks unless otherwise stated (20 – 30 g body weight). Mice were analyzed 12 (immunohistochemistry) and 20 days (electrophysiology) after tamoxifen induction.

### Immunostaining

After perfusion with 4% paraformaldehyde (w/v) in phosphate buffered saline (PBS, pH 7.4) for 20 min, tissue specimens were either cut on a vibratome (40 µm) or cryoprotected in 30% sucrose/PBS, frozen and cut on a cryostat at -22°C (spinal cord 14 µm, peripheral organs 20 µm). Tissue sections were processed for immunohistochemistry by permeabilization in 0.4% Triton X-100 (Sigma, T8787) in PBS for 30 min, blocking in 4% horse serum (HS) and 0.2% Triton X-100 in PBS for 30 min and incubation with first antibody in 1% HS and 0.05% Triton X-100 in PBS at 4°C overnight or for 48h (CAII and Olig2). Incubation with secondary antibodies and DAPI (4',6-diamidino-2-phenylindole) were in 1.5% HS in PBS for 2h at room temperature after which sections were mounted in AquaPolymount (Polysciences). Specimens were analyzed by epifluorescence microscopy using a Plan-Apochromat 20x/0.8 objective (Zeiss Axio Oberser.Z1 with ApoTome.2) and the ZEN 2 software (Zeiss). Confocal laser scanning microscopy (Leica SP2 equipped with a HC PL APO lambda blue 20x/0.7 objective or with a Leica SP5 (HCX PL APO CS 20x/0.7, HCX PL APO lambda blue 40x/1.25, HCX PL APO CS 100x/1.44 objectives) using the Leica Confocal Software (Leica Microsystems). Images were processed with NIH ImageJ and Adobe Photoshop CS5.1 softwares. For quantification, cells were counted on eight confocal images for each of the n=4 animals.

### Immunoblotting

Tissue was lysed in sucrose buffer containing 320 mM sucrose, 10mM Tris-HCL (pH 7.4), 1mM NaHCO _3_, 1mM MgCl _2_, 1% Triton X-100, 2% lithiumdodecylsulfate, 0.5% sodiumdeoxycholate, and protease and phosphatase inhibitors (cOmplete™, PhosSTOP™, Roche). 25 µg (brain tissue) and 20 µg (lung, liver, kidney, small intestine, heart) of protein lysates were resolved on 12% SDS-polyacrylamide gels under denaturing conditions and electro-transferred to PVDF membranes (Hybond P; GE Healthcare). Blocking was performed for 1h in Tris buffered saline / 0.05% Tween 20 (TBST) containing 5% milk powder and incubated in primary antibody at 4°C overnight in the same solution. Membranes were washed in TBST prior to incubation with appropriate horseradish peroxidase (HRP)-conjugated secondary antibodies (1:5000 Dianova, Hamburg) for 1h. Blots were developed by enhanced chemiluminescence (Pierce, Rockford) and scanned using the ChemoCam Imager (Intas Science Imaging Instruments, Goettingen).

### Antibodies

The following primary antibodies were used in this study: S100beta (rabbit monoclonal, 1:200, Abcam, ab52642), NSE (rabbit polyclonal, 1:500, Chemicon, AB951), CAII (polyclonal rabbit, 1:100, generous gift from S. Ghandour), GFAP (monoclonal mouse, 1:200, Chemicon, MAB3402), Parvalbumin (polyclonal rabbit, 1:1000, Swant, PV-28), Iba1 (rabbit polyclonal, 1:1000, Wako, 019-19741), Olig2 (polyclonal rabbit, 1:100, generous gift from Charles Stiles and John Alberta), RFP (polyclonal rabbit, 1:500 (immunostaining) or 1:1500 (immunoblotting), Rockland, 600-401-379), GAPDH (monoclonal mouse, 1:2500, Stressgen, CSA-335), and GFP (polyclonal goat, 1:500, Rockland, 600-101-215). We used Alexa Fluor 488-conjugated (1:2000, Invitrogen, A21206, 21202, A11055), Alexa Fluor 555-conjugated (1:2000, Invitrogen, A31572) and DyLight 633-conjugated (1:500, YO Proteins 356) secondary antibodies.

### Electrophysiology

Acute forebrain slices from 8 weeks old Aldh1l1-CreERT2*ROSA26-Tdto (n=3) mice were prepared as described previously ( Schnell *et al.*, 2015). Briefly, after deep isoflurane narcosis, animals were decapitated, the forebrain was prepared and placed in ice-cooled, carbogen-saturated (95 % O _2_, 5 % CO _2_) artificial cerebrospinal fluid (aCSF; in mM: 118 NaCl, 3 KCl, 1.5 CaCl _2_, 1 MgCl _2_, 1 NaH _2_PO _4_, 25 NaHCO _3_, and 30 D-glucose; 330 mosmol/l, pH7.4). Sagittal sections (300 µm) were cut on a vibroslicer (VT1200 S, Leica) and stored in aCSF at (35–36°C) for at least 30 min. Subsequently, slices were transferred to the recording chamber and kept submerged by a platinum grid with nylon fibers for mechanical stabilization. The chamber was mounted on an upright microscope (Axioscope FS, Zeiss Germany, 40x objective) and continuously perfused with aCSF at room temperature at a flow rate of 5–10 ml/min. Astrocytes were identified by their red fluorescence in epifluorescence illumination (white-LED, Lumencor Sola SE II) using a tdTomato optimized filter set (excitation 560/40 nm; dichroic mirror 595 nm, emission 645/75 nm; AHF Analysentechnik). For documentation, images of recorded tdTomato-expressing cells were taken with a CCD camera (Sensicam, PCO) and Imaging workbench 6.0 software (Indec Biosystems). Whole-cell voltage-clamp recordings were obtained with a MultiClamp 700B Amplifier (Molecular Devices). Patch electrodes were pulled from borosilicate glass capillaries (Biomedical Instruments, Zülpich, Germany) using a horizontal pipette-puller (Zeitz-Instrumente, Germany). Electrodes were filled with (in mM) 125 KCl, 1 CaCl _2_, 2 MgCl _2_, 4 Na _2_ATP, 10 EGTA, 10 HEPES (pH adjusted to 7.2 with KOH) leading to tip resistance of 2 – 6 MΩ. Currents were low-pass filtered at 3 kHz, and sampled at 10 kHz and recorded with pClamp 10 software (Molecular Devices) and stored for off-line analysis. Astrocytes were voltage-clamped to –80 mV and characterized by a voltage step protocol. Therefore, cells were hyperpolarized by -80 to -10 mV and depolarized by +10 mV to +110 mV voltage steps (10 mV increment).

## Data availability

The data referenced by this article are under copyright with the following copyright statement: Copyright: © 2016 Winchenbach J et al.

Data associated with the article are available under the terms of the Creative Commons Zero "No rights reserved" data waiver (CC0 1.0 Public domain dedication). 

**Dataset 1:** Raw data generated or analyzed during the present study in a zipped file. DOI, 10.5256/f1000research.10509.d147854 ( Winchenbach *et al.*, 2016).
