# Supplementary material for: Inducible targeting of CNS astrocytes in Aldh1l1-CreERT2 BAC transgenic mice
Source: F1000Res. 2016 Dec 30;5:2934. [Version 1] doi: 10.12688/f1000research.10509.1 (PMC5265728; doi:10.12688/f1000research.10509.1)
Supplement: Peer review history

# Peer review: Referee response for version 1

**DOI:** 10.5256/f1000research.11327.r18885

**Published:** 2017-02-20

**Authors:** Jeffrey D. Rothstein, Sean Miller

**Affiliations:** 1 Brain Science Institute, Depts of Neurology and Neuroscience, Johns Hopkins University, Baltimore, MD, USA; 2 Brain Science Institute, Johns Hopkins University, Baltimore, MD, USA

This manuscript is about the generation of a Aldh1l1-CreERT2 mouse model to better study astrocytes in vivo. Due to the lack of Cre-driven astrocyte-specific rodent models, manipulating and studying astrocytes has been exceptionally challenging. This new mouse model may open up many opportunities for scientists in the study of astrocytes. However, for the staining of astrocytes during cell counts and co-labeling the S100beta staining seems to label only a fraction of all astrocytes compared to reporter mouse models such as the BAC-Glt1-eGFP or Aldh1l1-eGFP; an antibody that could detect all astrocytes would be the best to quantify overall double positive astrocytes such antibody combinations of Glt1, Aldh1l1, and/or Acsbg1. In an ideal situation you would generate a triple transgenic with the Aldh1l1-CreERT2, Rosa26-Tdtomato, and BAC-Glt1-eGFP to obtain more accurate cell counts and co-labeling. Although this experiment would take considerable time to complete and might be a fine followup study. Overall, this study will provide a greatly needed tool to glial biology. Provided these approaches such as staining are considered, we approve this manuscript for indexing.

We have read this submission. We believe that we have an appropriate level of expertise to confirm that it is of an acceptable scientific standard.

# Peer review: Referee response for version 1

**DOI:** 10.5256/f1000research.11327.r18882

**Published:** 2017-01-23

**Authors:** Andrea Volterra, Iaroslav Savtchouk

**Affiliations:** 1 Department of Fundamental Neurosciences, University of Lausanne, Lausanne, Switzerland; 2 University of Lausanne, Lausanne, Switzerland

The paper by Winchenbach *et al*. describes a newly generated astrocyte-specific Aldh1L1-CreERT2 mouse line resulting in Cre-mediated recombination after Tamoxifen injection.

 This new mouse line addresses the crucial need in the field for a conditional line in which Cre is both astrocyte-specific, and yet present in the absolute majority of astrocytes homogeneously throughout the CNS. Prior models have reportedly suffered from either lower astrocyte specificity (such as certain Glast-CreERT lines), or lower levels of astrocytic expression, with significant differences depending on the brain area (such as in GFAP-CreERT2 lines). The authors commit to making their new mouse line freely available on request.

 On balance, the new Aldh1l1-CreERT2 mouse line from Nave/Saher, together with a similar line independently generated and recently published by the Khakh lab ^1^ have a large potential to advance astroglial research. Both labs have done a great service to the field by making their mice freely available. Consequently, these are likely to be highly useful tools for generating and studying astrocyte-specific knockouts and even knock-ins.

 Detailed comments:

 In this paper the authors used 5 Tamoxifen injections to induce recombination in Aldh1l1-CreERT2 animals crossbred with either Rosa-26-tdTomato or -YFP mice, and quantified the % recombination in S100beta-positive cells (putative astrocytes) across different brain regions after 7 days by immunohistochemistry. A subset of cells was additionally examined after 20 days by electrophysiology. Other controls were also performed.

 Co-staining with neuronal markers shows very limited overlap with neurons; recombination in a small (3%) subset of parvalbumin-positive cells of the cerebellum is judged to be the artifact of the tdTomato reporter line as it was absent in the YFP line. Neuronal expression in areas of adult neurogenesis is also reported, similar to what was already observed in prior astrocyte lines. Peripheral organs were also examined, and the authors report expected recombination in some of them.

 The manuscript is technically sound and of high scientific quality. The paper provides a thorough characterization of the new line which has the potential to be highly useful for the field.

 Below we list some minor suggestions for improving the paper, as well as some general points applicable for the entire field.

 Issues to be addressed for Aldh1l1-CreERT2 mice:

1. The authors could add more information regarding image quantification (Tables 1 & 2). Do “eight confocal pictures” mean single-plane images of different areas of the slice, or stacks? Of what thickness? What was the zoom, axial/lateral resolution, field of view?
2. The authors provide a helpful electrophysiological confirmation of reporter-expressing cells as passive astrocytes. As a very minor methodological point, they could indicate whether they used liquid junction potential correction.
3. **Definitive marker for “astrocytes.”** Can the authors discuss why they think that S100Beta is the best marker of astrocytes? This is relevant in particular for correctly interpreting the existing  S100Beta-negative population as astrocytic or non-astrocytic: indeed, the authors report that about 4-19% of S100Beta-negative cells also express reporters (Table 1). What are those cells?
4. Related to the previous point, the authors have performed GFAP co-staining but do not currently report the % co-labelling due to difficulty in quantification. An image from spinal cord is shown in Fig. 2d. However, some summary statement, even based on a limited number of manually analyzed cells, would be helpful. Do they see some of the S100beta-negative (reporter-positive) cells also positive for GFAP?
5. The authors report about 6% of cells double-label for markers of other cell types (e.g. oligodendrocyte precursors or microglia) in Table 2. Do these account for the 4-19% of S100Beta-negative/reporter-positive cells in Table 1? Importantly, neuronal co-labelling is reported as nonexistent (with NSE) at least in the cortex. Is this the same also in other areas?
6. With the publication of the present line, there are now two Aldh1l1-CreERT2 mouse lines openly available on the “market.” It remains for the field to determine which of the two lines has the most reliable and therefore useful profile, meaning  highest astrocyte specificity as well as  highest recombination efficiency in astrocytes. As the authors suggest, there may already be some differences regarding expression in e.g. NG2 cells, to be determined in the follow-up studies.
7. Preserving a “reference” strain of mice (e.g. via frozen sperm) may be helpful to avoid gene drift and future emergence of colonies with different properties. Ostensibly, this mechanism may explain conflicting results historically reported for other “astrocytic” mice in the field such as dnSNARE (see e.g. Fujita *et al.* J Neurosci 2014, reviewed in Bazargani and Attwell, Nat. Neurosci. 2016)
8. “Titration” curve for tamoxifen induction **.** One of the strengths of this mouse line is the very high recombination efficiency across diverse astrocyte populations: ~90% of likely astrocytes after just five Tamoxifen injections. The authors emphasize the need to determine the % recombination for each individual line. Additionally, it would be useful in the future to know how differences in tamoxifen treatment regime correspond to different levels of recombination for some common lines (e.g. Rosa26-tdTomato). For instance, is a single injection sufficient to cause recombination in the bulk of astrocytes? Is it also possible to achieve sparser expression of the reporter (for single-cell imaging studies) with a reduced Tamoxifen administration (e.g. single-day)? Can the % recombination be raised over 90%, and if so, after how many injections? Obviously, the ultimate % recombination will depend also on a chosen reporter line (as apparent from Table 1), but more preliminary information for common lines would already be helpful.

We have read this submission. We believe that we have an appropriate level of expertise to confirm that it is of an acceptable scientific standard.

## References

[1] SrinivasanRLuTYChaiHXuJHuangBSGolshaniPCoppolaGKhakhBS: New Transgenic Mouse Lines for Selectively Targeting Astrocytes and Studying Calcium Signals in Astrocyte Processes In Situ and In Vivo. *Neuron*.2016;92(6) : 10.1016/j.neuron.2016.11.030 1181-1195 10.1016/j.neuron.2016.11.030 27939582PMC5403514

# Peer review: Referee response for version 1

**DOI:** 10.5256/f1000research.11327.r19456

**Published:** 2017-01-18

**Authors:** David H. Rowitch

**Affiliations:** 1 Department of Paediatrics, Wellcome Trust-Medical Research Council Cambridge Stem Cell Institute, University of Cambridge, Cambridge, UK

This manuscript by Winchenbach *et al* described generation and characterization of a new transgenic tool to investigate astrocyte biology.  The field is currently limited by cre recombinase driver transgenic reagents that show poor coverage and/or temporal control during central nervous system development.  The current paper uses ALDH1L1 cre ERT2 BAC transgenic mice to address limitations in the field, developing a useful new tool.  The data presented are technically sound and included the use of crosses with two conditional reporter alleles, single cell patch clamp electrophysiological analysis to confirm astrocyte features and inclusionary and exclusionary immunohistochemistry.  The conclusions are supported by data and also highlight the utility of this new transgenic allele, compared to a commonly-used (GLAST-CRE) transgenic driver mouse, such that I think will be of significant interest in this new mouse line from the glial biology community.

I have read this submission. I believe that I have an appropriate level of expertise to confirm that it is of an acceptable scientific standard.
